# Changes in thrombosis-related parameters after AstraZeneca COVID-19 vaccination in a male volunteer: a case report

**DOI:** 10.1186/s13256-022-03563-9

**Published:** 2022-08-23

**Authors:** Sae-Yong Hong, Sang-Sin Jun, Sang-Wook Seo, Jeong-Rae Park, Joung-Il Im

**Affiliations:** 1 Department of Nephrology, Jincheon Joongang Jeil General Hospital, 24 Jungang-bukro, Jincheoneup, Jincheongun, Chungbuk, 27832 Korea; 2 Department of Neurology, Jincheon Joongang Jeil General Hospital, Chungbuk, Korea; 3 Department of Family Medicine, Jincheon Joongang Jeil General Hospital, Chungbuk, Korea; 4 Department of Clinical Pathology, Jincheon Joongang Jeil General Hospital, Chungbuk, Korea; 5 Department of Orthopedic Surgery, Jincheon Joongang Jeil General Hospital, Chungbuk, Korea

**Keywords:** COVID-19 vaccine, Adverse drug reaction, Blood coagulation, Fibrinolysis, Case report

## Abstract

**Background:**

We speculated that subclinical thrombosis may occur frequently through crosstalk between immune/inflammatory reactions and hemostasis after corona virus disease-2019 (COVID-19) vaccination. To test this hypothesis, we measured thrombosis-related parameters after COVID-19 vaccination in a volunteer for 21 days.

**Case presentation:**

The following parameters were measured in a 72-year-old Korean man at 1 day before vaccination and on days 1, 3, 7, 14, and 21 post vaccination (AstraZeneca COVID-19 vaccine: ChAdOx1-S/nCoV-19, CTMAV563): complete blood count, platelet indices, thrombin receptor-activating peptide-induced platelet aggregation, prothrombin time, activated partial thromboplastin time, D-dimer, thrombin–antithrombin III complex (TAT), plasmin-α2 antiplasmin complex (PAP), von Willebrand factor (vWF) antigen and activity, plasminogen activator inhibitor-1 (PAI-1), protein C and protein S antigen and activity, lupus anticoagulant, fibrinogen degradation product, and plasminogen. We found that the TAT had significantly increased from 0.7 ng/mL (baseline) to 21.7 ng/mL (day 1). There was a transient increase in the PAI-1 level from 7.2 ng/mL (baseline) to 10.9 ng/mL (day 3), followed by a decrease in PAP level from 0.9 ng/mL (baseline) to 0.3 μg/mL (day 7), suggesting that plasmin generation is suppressed by PAI-1.

**Conclusions:**

Increased thrombotic factors (such as decreased protein S) and decreased fibrinolytic activity due to increased PAI-1 were potential factors causing thrombogenesis after COVID-19 vaccination. Sequential measurement of platelet indices, TAT, PAP, protein C, protein S, vWF, D-dimer, and PAI-1 following COVID-19 vaccination was informative.

## Background

Coronavirus disease–2019 (COVID-19) vaccine-associated immune thrombosis and thrombocytopenia (VITT) have emerged as serious adverse effects of COVID-19 vaccines, particularly vector vaccines, such as those manufactured by Oxford/AstraZeneca and Janssen/Johnson & Johnson [1, 2]. However, it is uncertain whether VITT is the only thrombosis-related disease that occurs after COVID-19 vaccination. We speculated that there may be many more unaccounted instances of subclinical thromboses, which may be triggered through crosstalk between hemostasis and inflammation/immune reactions via the release of various cytokines after COVID-19 vaccination [3].

To test our hypothesis, we measured thrombosis-related parameters sequentially for 21 days after COVID-19 vaccination in a male volunteer.

## Case presentation

The volunteer was a 72-year-old Korean man (weight 62.5 kg; height 168 cm). Vital signs on the day before vaccination were: blood pressure, 120/80 mmHg; pulse rate, 70 beats/min; and body temperature, 36.8 °C. He was diagnosed with diabetes mellitus, hypertension, and hypercholesterolemia at the age of 45 years. His routine medications were atorvastatin (10 mg), aspirin (100 mg), diltiazem (180 mg), and long-acting insulin, which continued throughout the observation period. The baseline laboratory findings are presented in Table 1.

### Timeline

At 3 p.m. on 9 June 2021, the volunteer received the AstraZeneca COVID-19 vaccine (5 mL) in the left deltoid muscle (ChAdOx1-S/nCoV-19 [recombinant] vaccine, CTMAV563). Blood samples were obtained 1 day before vaccination (baseline) and on days 1, 3, 7, 14, and 21 post vaccination.

### Diagnostic assessment

The following parameters were measured in the context of routine testing in a selected general hospital: platelet indices (PI), including mean platelet volume (MPV) and platelet distribution width (PDW), and platelet-large cell ratio (P-LCR) were measured together on a fully automated hematology analyzer (Sysmex XN-1000^TM^; Sysmex Corp., Kobe, Japan). Thrombin receptor-activating peptide (TRAP)-induced platelet aggregation was measured using a Multiplate® analyzer (Roche Diagnostics GmbH, Mannheim, Germany). Prothrombin time, activated partial thromboplastin time, and D-dimer were measured on the STACompact Max® analyzer (Diagnostica Stago, Asnieres, France).

Thrombin–antithrombin III complex (TAT) and plasmin-α2 antiplasmin complex (PAP) assays were outsourced to BioMedical Laboratories, Japan (BML Japan: http://www.bml.co.jp). The assay kits used were the HISCL® TAT test kit (Sysmex Corp.) for TAT assessment and the LPIA-NV7 STACIA® test kit (LSI Medicine Corp., Tokyo, Japan) for PAP assessment.

Assays for the following parameters were outsourced to EONE Laboratories (Incheon South Korea: https://www.eonelab.co.kr/institution/certi_list.asp): both von Willebrand factor (vWF) antigen and activity (Instrumentation Laboratory, Bedford, MA, USA); plasminogen activator inhibitor-1 (PAI-1; Asserachrom PAI-1 enzyme immunoassay kit; Diagnostica Stago); protein C and protein S antigen levels (enzyme-linked immunosorbent assay kit; Corgenix Medical Corp., Broomfield, Colorado, USA); protein C activity (synthetic chromogenic substrate method; Stachrom protein C kit; Diagnostica Stago); protein S activity (Factor Va inhibition test kit; Diagnostica Stago); lupus anticoagulant (diluted Russell viper venom test; Diagnostica Stago); fibrinogen degradation product (FDP; antibody-coated latex agglutination kit; Sekisui Medical Co. Ltd., Tokyo, Japan); and plasminogen (synthetic chromogenic substrate method; Stachrom Plasminogen kit; Diagnostica Stago).

## Discussion and conclusions

It is widely acknowledged that the generation of platelet factor 4 (PF4)-polyanion antibodies is the essential pathophysiological mechanism underlying VITT development after COVID-19 vaccination [4]. However, the presence of this antibody does not always indicate thrombosis after COVID-19 vaccination [5]. What is the mechanism of PF4-polyanion antibody-negative thrombosis after COVID-19 vaccination? What kind of changes could take place in thrombosis-related parameters after COVID-19 vaccination in the general population? To answer these questions, we measured thrombosis-related parameters, before and after COVID-19 vaccination, sequentially for 21 days.

**Fig. 1:**
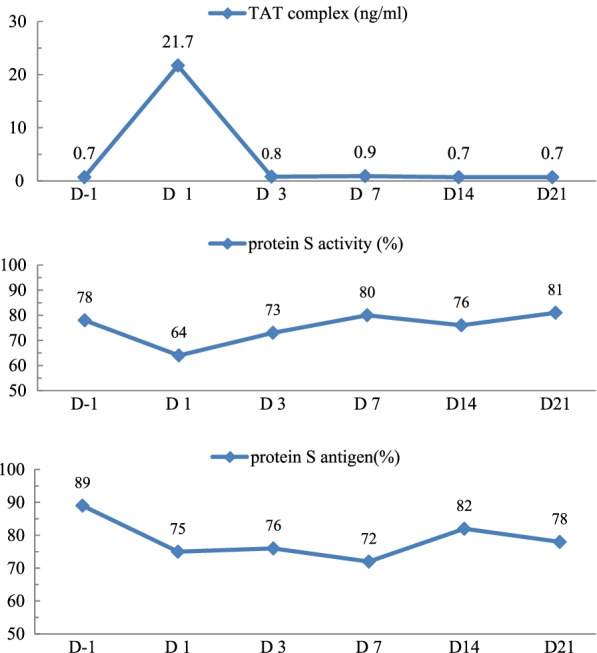
Sequential measurement of thrombin–antithrombin III complex (*TAT*), protein S activity, and protein S antigen. The reference range of TAT in the general population has been reported to be < 13.0 ng/mL (see text). Note that TAT levels increased markedly on day 1 post vaccination, but protein S activity and antigen levels decreased during the first few days. The reference range in the general population, provided by the assay kit manufacturer, is 60–140% for protein S activity and 60–150% for protein S antigen

**Fig. 2:**
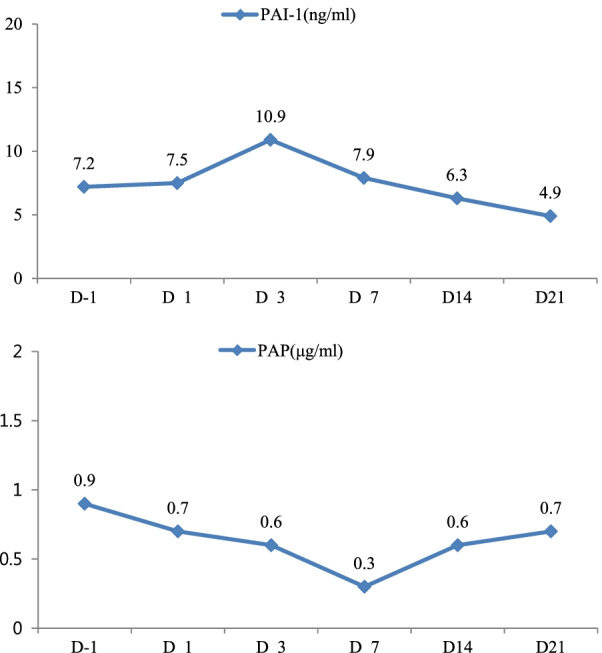
Sequential measurement of plasmin-α_2_ antiplasmin complex (*PAP*) and plasminogen activator inhibitor-1 (*PAI-1*) levels. As per the manufacturing information on the assay kits, the reference level in healthy donors is < 0.8 μg/mL for PAP and 4.0–43.0 ng/mL for PAI-1. Note that the PAP levels decreased markedly on day 7 post vaccination and that PAI-1 levels decreased on day 3 post vaccination

The volunteer experienced no abnormalities, except mild fever 2 days after vaccination. However, various thrombotic parameters changed during the first few days after receiving the COVID-19 vaccination (Table 1; Figs. 1, 2).

**Table 1:** Sequential measurement of thrombosis-related parameters after coronavirus disease-2019 vaccination

Parameters^a^	Baseline	Sampling day post vaccination				
		1	3	7	14	21
*Platelet indices*						
Platelet count (10^3^/μL)	168	147	159	153	168	169
PDW(fL)	8.7	7.9	8.8	8.5	9.6	8.7
MPV(fL)	8.9	8.6	8.9	8.8	9.0	9.2
P-LCR (%)	15.7	14.1	16.3	15.5	17.6	17.7
Plateletcrit (%)	0.15	0.13	0.14	0.14	0.15	0.16
TRAP aggregation (AUC unit)	101	115	104	108	104	93
*Coagulation*						
Prothrombin time (seconds)	13.3	13.6	13.4	13.3	13.0	13.8
Prothrombin time (%)	99	95	98	99	97	86
Prothrombin time (INR)	1.00	1.03	1.01	1.00	1.02	1.10
APTT (seconds)	36.0	37.1	35.8	36.3	39.1	37.5
vWF activity (%)	185	162	147	160	157	162
vWF antigen (%)	185	183	179	176	168	169
Protein C activity (IU/dL)	91	88	91	92	87	86
Protein C antigen (IU/dL)	69.2	70.4	77.5	81.5	71.6	66
Lupus anticoagulant	–	–	–	–	–	–
*Fibrinolysis*						
Plasminogen (%)	89	86	91	92	85	87
FDP (μg/mL)	< 0.25	< 0.25	< 0.25	< 0.25	< 0.25	< 0.25
D-dimer (μg/mL)	0.31	0.35	0.41	0.40	0.34	0.35

A blood sample for determining baseline values was obtained 1 day before vaccination

* PDW* platelet distribution width, * MPV* mean platelet volume, * P-LCR* platelet-large cell ratio, * TRAP* thrombin receptor-activating peptide,* AUC* area under the curve, *INR* international normalized ratio, * APTT* activated partial thromboplastin time,* vWF* von Willebrand factor, * FDP* fibrinogen degradation product

^a^Reference normal range (in parentheses): platelet count (130–450 10^3^/μL), PDW (10.0–20.0 fL), MPV (7.0–13.0 fL), P-LCR (15–35%), TRAP (92.0–151.0 AUC unit), prothrombin time (11.5–15.0 seconds), prothrombin time (70.0–135.0%), prothrombin time (0.84–1.21 INR), APTT (28.0–45.0 seconds), vWF activity (45.6–176.3%), vWF antigen (50.0–160.0%), FDP (0–5.0 μg/mL),* D-dimer* (0–0.63 μg/mL), protein C activity (72.0–160.0 IU/dL), protein C antigen (73.0–142.0 IU/dL), plasminogen (80.0–120.0 %)

The most remarkable findings were the changes in both TAT and PAP, which indicate the generation of thrombin and plasmin, respectively [6]. The TAT level increased markedly on day 1 post vaccination from 0.7 (baseline) to 21.7 ng/mL (Fig. 1). Both the antigen level and activity of protein S decreased on days 1–3 following vaccination. As a cofactor of protein C, protein S inactivates coagulation factors Va and VIIIa. Therefore, we speculate that the AstraZeneca COVID-19 vaccine suppresses protein S, consequently provoking the generation of thrombin in circulation.

Both vWF antigen and activity decreased transiently following vaccination (Table 1; the reference ranges for the general population, provided by the assay kit manufacturer, are 45.6–176.3% for vWF antigen and 50.0–160.0% for vWF activity). During the first few days, protein S and vWF appeared to conflict with each other in the thrombotic process, given that decreased protein S is a thrombogenic factor, while decreased vWF is an antithrombotic factor. However, increased levels of D-dimer are a surrogate marker of thrombus formation, suggesting that thrombotic activity outweighed antithrombotic activity for the first few days following COVID-19 vaccination.

There was a transient increase in PAI-1 levels from 7.2 (baseline) to 10.9 ng/mL (day 3) (Fig. 2), followed by a decrease in PAP levels from 0.9 (baseline) to 0.3 μg/mL (day 7), suggesting that plasmin generation is suppressed by PAI-1 (Fig. 2). The D-dimer level increased slightly on days 3 and 7, while the FDP level remained below the cut-off level of the assay kit (< 0.25 μg/mL) throughout the observation period. Considering the results in this case, we surmise that the fibrinolysis was not stimulated by the vaccine itself, and the reason for the increased D-dimer was a physiological response to fibrin (thrombus) formation, rather than enhanced fibrinolytic activity.

There was a transient reduction in platelet counts, from 168 × 10^3^ to 147 × 10^3^/μL at 1 day after COVID-19 vaccination (Table 1). A platelet count < 150 × 10^3^/μL is considered as ‘thrombocytopenia,’ which is caused by either decreased production, increased destruction, or increased trapping of platelets in the spleen. We could not identify the etiology of thrombocytopenia in this case.

The volunteer was an elderly person (72 years old) with diabetes mellitus, hypertension, and hypercholesterolemia. Therefore, all the previously mentioned points, such as age and medical history, may be risk factors of thrombosis. However, we believe that if the comorbidities were the etiology underlying the change in the coagulation profile, the change would continue before and after vaccination.

As seen with platelet counts, MPV, PDW, and P-LCR were lower on post-vaccination day 1 than at baseline. Furthermore, TRAP-induced platelet aggregation increased from 101 area-under-the-curve (AUC) units to 115 AUC units after COVID-19 vaccination (Table 1), suggesting that platelet aggregation was stimulated by the COVID-19 vaccine. Further research is necessary to evaluate changes in platelet function during the first few days following COVID-19 vaccination.

This study has its limitations. This report only describes the case of a single volunteer, and we only measured selective thrombotic and antithrombotic parameters. Therefore, it is uncertain whether our observations can be applied to other cases. However, even with this limitation, our observation indicates that various thrombotic parameters may change during the first few days after COVID-19 vaccination, without signs of clinically overt thrombosis.

Increased thrombotic factors (such as decreased protein S) with decreased fibrinolytic activity due to increased PAI-1 seem to be potential factors causing thrombogenesis after COVID-19 vaccination. To better understand the progression of thrombosis, sequential measurement of PI, TAT, PAP, protein C, protein S, vWF, D-dimer, and PAI-1 after COVID-19 vaccination seems to be informative.

## Data Availability

Not applicable.

## Abbreviations

COVID-19: Coronavirus disease 2019
FDP: Fibrinogen degradation product
MPV: Mean platelet volume
PAI-1: Plasminogen activator inhibitor-1
PAP: Plasmin-α2 antiplasmin complex
PDW: Platelet distribution width
PF4: Platelet factor 4
PI: Platelet indices
P-LCR: Platelet-large cell ratio
TAT: Thrombin–antithrombin III complex
TRAP: Thrombin receptor-activating peptide
VITT: Vaccine-associated immune thrombosis and thrombocytopenia
vWF: Von Willebrand factor
